# Prevalence of ESBL-associated resistance genes and antimicrobial resistance in *Escherichia coli* from pigs and cattle slaughtered at urban abattoirs in Lilongwe District, Malawi

**DOI:** 10.3389/fvets.2026.1886903

**Published:** 2026-09-10

**Authors:** Charles Nkhoma, Naomi Chikwana, Precious Innocent Mastala, Thoko Kapalamula

**Affiliations:** 1 Department of Animal Health and Livestock Development, Central Veterinary Laboratory, Lilongwe, Malawi; 2 Department of Basic Sciences, Lilongwe University of Agriculture and Natural Resources (LUANAR), Lilongwe, Malawi; 3 Department of Epidemiology and Biostatistics, Mel and Enid Zuckerman College of Public Health, University of Arizona, Tucson, AZ, United States; 4 Department of Pathobiology, Faculty of Veterinary Medicine, LUANAR, Lilongwe, Malawi

**Keywords:** abattoir, ESBL, *Escherichia coli*, Malawi, multidrug resistance, One Health

## Abstract

Extended-spectrum beta-lactamase (ESBL)-producing *Escherichia coli* in food-producing animals represents a priority public health concern because of its capacity for zoonotic transmission through the food chain and frequent co-carriage of multidrug resistance (MDR) determinants. Surveillance in sub-Saharan Africa remains disproportionately limited, and no published data characterize ESBL prevalence or resistance gene profiles in slaughter animals in Malawi. This study investigated the prevalence of *E. coli* harboring ESBL-associated resistance genes (*blaCTX-M*, *blaSHV*) and characterized antimicrobial resistance and MDR profiles in pigs and cattle slaughtered at urban abattoirs in Lilongwe District, Malawi. Rectal swabs were collected from 50 pigs and 50 cattle (*n* = 100) at four urban abattoirs. Following exclusion of six samples with no bacterial growth, 94 confirmed isolates were analyzed. *E. coli* was identified by MALDI-TOF MS. Antimicrobial susceptibility testing was performed by disk diffusion (CLSI) against gentamicin, chloramphenicol, ciprofloxacin, sulfamethoxazole-trimethoprim, cefotaxime, and tetracycline. *blaCTX-M* and *blaSHV* genes were detected by conventional PCR. Of 94 confirmed *E. coli* isolates (48 pig, 46 cattle), pig isolates showed significantly higher resistance to gentamicin (50.0% vs. 15.2%; *p* = 0.0008) and tetracycline (85.4% vs. 56.5%; *p* = 0.004). Chloramphenicol resistance was high in both species (77.1% pigs; 63.0% cattle). *blaCTX-M* was detected in 12.5% of pig and 21.7% of cattle isolates; *blaSHV* in 20.8 and 6.5%, respectively (all *p* > 0.05). MDR was significantly more prevalent in pig isolates (70.8%, 34/48) than in cattle isolates (41.3%, 19/46; crude OR = 3.40, 95% CI 1.35–8.90; *p* = 0.004). Pigs had a mean resistance count of 3.17 antibiotic classes per isolate compared with 2.26 in cattle. *E. coli* harboring targeted ESBL-associated genes circulates in both species at urban abattoirs in Lilongwe District, with pig isolates carrying a significantly higher MDR burden. These results provide an initial baseline for monitoring *E. coli* harboring targeted ESBL-associated genes at the livestock-food chain interface in Malawi. While stewardship practices were not directly assessed, the findings raise important implications for species-stratified antibiotic stewardship and highlight the value of systematic antimicrobial resistance monitoring within a One Health framework.

## 1 Introduction

Antimicrobial resistance (AMR) has emerged as one of the most consequential threats to global public health. In 2019 alone, AMR was associated with nearly five million deaths worldwide, with 1.27 million directly attributable to drug-resistant bacterial infections (1). Projections suggest that without coordinated intervention, AMR could cause an estimated 39.1 million deaths attributable to AMR cumulatively between 2025 and 2050 (2). Within the One Health framework, extended-spectrum beta-lactamase (ESBL)-producing *Escherichia coli* has emerged as a pathogen of particular concern (28). *E. coli* colonizes the gastrointestinal tracts of mammals as a commensal, but its genetic plasticity facilitates the acquisition and horizontal transfer of resistance determinants. ESBLs confer resistance to extended-spectrum cephalosporins and aztreonam through plasmid-borne genes that can spread across bacterial species and host–environment interfaces (3, 4). Intestinal carriage of ESBL-producing *E. coli* increased by an estimated 25% in humans across community and health care settings over the past two decades (30). The most clinically prevalent gene families *blaCTX-M* and *blaSHV* are now distributed globally, with *blaCTX-M* variants driving much of the recent expansion in community and livestock settings (5, 6).

Food-producing animals and slaughter systems represent critical interfaces in the transmission of ESBL-producing *E. coli* to humans. Antibiotic use under farming conditions creates strong selective pressure in the gut microbiome of livestock; resistant strains contaminate carcasses during slaughter and enter the human food supply through meat products. Disposal of gut effluent further disseminates resistant organisms into the wider environment, sustaining transmission cycles across human, animal, and ecological compartments (7). ESBL resistance patterns documented in food animals closely mirror those observed in human urinary tract infections and *Enterobacterales* bloodstream infections (8). Urban abattoirs are particularly important sentinel sites: they receive animals from geographically diverse farm sources, concentrate slaughterhouse workers as a high-exposure human population, and distribute meat products widely into urban markets. Recent studies from other regions illustrate the diversity of MDR *E. coli* circulating in livestock. In Algeria, ESBL-associated resistance genes together with integron-mediated resistance were reported in cattle (9). In China, the plasmid-mediated colistin-resistance gene *mcr-1* was identified in *E. coli* from pig farms in Jiangxi (10). A study of household cattle in the Mekong Delta, Vietnam, demonstrated multidrug resistance in *E. coli* (11), while fluoroquinolone-resistant *E. coli* genes were reported in Korean pig farms (12, 29). In Turkey, a study of cattle, sheep, and goats reported high rates of multiple *E. coli* serotypes and virulence subtypes, including enteroaggregative *E. coli* (EAEC, CVD432) and Shiga toxin-producing *E. coli* (STEC, stx1/stx2) (13). Collectively, these studies highlight the importance of MDR surveillance and reveal considerable variation in prevalence across geographical settings.

Despite extensive studies in Europe and Asia (5, 6, 14), surveillance data on ESBL-producing *E. coli* in sub-Saharan Africa remain disproportionately limited relative to the region’s AMR burden. In Malawi the National Action Plan for Antimicrobial Resistance was developed for 2018–2023, designating AMR as a priority area in accordance with the WHO Global Action Plan on Antimicrobial Resistance. A One Health approach integrating the animal, human, and environmental sectors in AMR surveillance is being progressively implemented in Malawi (15, 16); however, pigs and cattle slaughtered at urban abattoirs remain under-characterized in terms of.

ESBL-associated resistance genes in *E. coli*. Pigs and cattle represent contrasting production systems likely to shape resistance dynamics differently. Pigs in Malawi are predominantly reared under intensive or peri-urban systems with higher antibiotic exposure; cattle are more commonly raised under free-range or extensive systems with comparatively lower exposure (17). Characterizing *blaCTX-M* and *blaSHV* prevalence across both species together with phenotypic resistance profiles therefore provides a comparative lens on how farming intensity influences the ESBL reservoir at the point of slaughter.

This study was therefore designed to: (1) determine the prevalence of *blaCTX-M* and *blaSHV* genes in *E. coli* isolates from pigs and cattle at urban abattoirs in Lilongwe District; (2) characterize antimicrobial resistance phenotypes across six antibiotic classes; (3) quantify the multidrug resistance (MDR) burden by species; and (4) evaluate factors associated with MDR relevant to One Health stewardship.

## 2 Materials and methods

### 2.1 Study design, population, and location

A cross-sectional study was conducted between April and June 2024 at four urban abattoirs within Lilongwe District in Malawi, under the Lilongwe Agricultural Development Division (LADD): Mkwinda, Chankhandwe, Lilongwe Cold Storage, and Chinsapo. The study targeted clinically healthy pigs and cattle presented for routine slaughter, and a total of 100 animals were sampled, with 25 animals from each abattoir. Malawi Zebu cattle and Large White pigs were the breeds sampled. Eligible animals were clinically healthy pigs and cattle presented for routine slaughter on designated sampling days; the number of animals available for sampling on a given day was determined by each abattoir’s slaughter throughput. Sampling days for cattle at Lilongwe Cold Storage were Wednesday, Thursday, and Friday of the first week of April 2024, and for Chankhandwe abattoir the same slaughter days were sampled during the third week of April 2024. Animals slaughtered at Lilongwe Cold Storage and Chankhandwe originate from multiple districts of Malawi, including Lilongwe, Dedza, Kasungu, Dowa, Mchinji, Salima, Nkhotakota, Nsanje, Zomba, Blantyre, Mangochi, and Mzimba, representing the Central, Southern, and Northern regions of Malawi. For pigs, slaughter activities occur on Wednesdays and Saturdays at Chinsapo and sampling was carried on the first 3 weeks of May 2024 while for Mkwinda slaughters occurs on Thursday and sampling was conducted during the mid-two weeks of June 2024. No refusals or replacements occurred during the sampling process for any of the animals.

### 2.2 Sample size and sampling technique

The sample size was calculated using the single population proportion formula:

$$n=[z^{2}\times \hat{p}(1–\hat{p})]/\varepsilon^{2}$$

where n = required sample size; z = Z-score for the 95% confidence level (1.96); p̂ = estimated proportion of ESBL-producing *E. coli* (0.5); and ϵ = margin of error (0.10). This formula was used to determine the sample size needed to estimate the true proportion of ESBL-producing *E. coli* in the study population with ±10% precision at 95% confidence. Because no prior prevalence data existed for ESBL-producing *E. coli* in food animals in Malawi, *p̂* = 0.5 was selected as the most conservative assumption, maximizing the variance term and producing the largest possible required sample size regardless of the true prevalence. This formula yielded a minimum of 96 animals; the final target was set at 100 (50 pigs, 50 cattle). A margin of error of 10% was chosen to balance statistical reliability with logistical constraints. Equal allocation of 50 animals per species was deliberate, enabling direct interspecies comparison. The cap of 25 animals per abattoir ensured representation across all four sites. Animals were selected by lottery-based simple random sampling from those presented for slaughter at each abattoir on sampling days.

### 2.3 Sample collection and processing

Rectal swabs were collected from each animal immediately prior to slaughter using sterile swabs with transport media, as described by Eriksen et al. (18) and Choudhury et al. (19). Swabs were placed in a cooler box and transported to the Central Veterinary Laboratory in Lilongwe under cold-chain conditions for same-day processing.

### 2.4 Isolation and identification of *E. coli*

Rectal swabs were pre-enriched in 20 mL buffered peptone water at 37 °C for 18–24 h. A loopful was sub-cultured onto MacConkey agar and blood agar and incubated at 37 °C for 24 h. Pink, lactose-fermenting colonies consistent with *E. coli on* MacConkey agar were selected from each swab based on colony morphology as described by Lupindu (20) subjected to MALDI-TOF MS (MALDI Biotyper 3.0 RTC database, Bruker Daltonics) for species confirmation, following the approaches of Singh et al. (8).

### 2.5 Antimicrobial susceptibility testing

Antimicrobial susceptibility testing (AST) was performed by the Kirby–Bauer disk diffusion method on Mueller–Hinton agar according to CLSI breakpoints. Six antimicrobials were tested: gentamicin (GM, 10 μg), chloramphenicol (CHL, 30 μg), ciprofloxacin (CIP, 5 μg), sulfamethoxazole-trimethoprim (SXT, 25 μg), cefotaxime (CTX, 30 μg), and tetracycline (TET, 30 μg). Bacterial suspensions were prepared to 0.5 McFarland standard. *E. coli* ATCC 25922 (ESBL-negative) and *Klebsiella pneumoniae* ATCC 700603 (ESBL-positive) served as quality controls. MDR was defined as resistance to three or more antibiotic classes tested.

### 2.6 Genotypic detection of ESBL genes

Out of the 100 samples which were collected, six did not yield bacterial colonies during bacterial culture and therefore were excluded from downstream analysis. Prevalence estimates were therefore calculated using the remaining 94 isolates as the denominator. Genomic DNA was extracted by heat lysis as described by Dashti et al. (21) and Lestari et al. (22). PCR was performed using ExTaq HS (TaKaRa, Japan) in a 25 μL reaction volume targeting *blaCTX-M* and *blaSHV* genes (Table 1) but this does not confirm phenotype and disk diffusion was conducted in parallel to characterize phenotypic resistance profiles. Primer sequences used to detect ESBL associated resistance genes were: for *bla*SHV, primers SHV-F (5′-GGTTATGCGTTATATTCGCC-3′) and SHV-R (5′-TTAGCGTTGCCAGTGCTC-3′) were employed, yielding an amplicon of 865 bp. For *bla*CTX-M, primers CTX-MA1 (5′-SC SATGTGCAGYACCAGTAA-3′) and CTX-MA2 (5′-CCGCRATATGRTTGGTGGTG-3′) were used, producing a 544 bp fragment. Cycling conditions for *blaCTX*-M: 94 °C/1 min initial denaturation; 35 cycles of 98 °C/30 s, 62 °C/30 s, 68 °C/1 min; final extension 68 °C/5 min. Conditions for *blaSHV* were identical except 40 cycles were used. PCR products were analyzed by 1.5% agarose gel electrophoresis with a 100 bp ladder. The isolates which were reported negative for these targeted genes in this study they may still harbor other ESBL associated resistance genes, AmpC, or β lactamase β-lactamase resistance determinants.

**Table 1:** Primer sequences used for ESBL gene detection by conventional PCR.

Primer	Target gene	Sequence (5′–3′)	Amplicon (bp)
SHVF/SHVR	*blaSHV*	GGTTATGCGTTATATTCGCC/TTAGCGTTGCCAGTGCTC	865
CTX-MA1/CTX-MA2	*blaCTX-M*	SCSATGTGCAGYACCAGTAA/CCGCRATATGRTTGGTGGTG	544

bp, base pairs.

### 2.7 Statistical analysis

All statistical analyses were performed in R version 4.3.3 (23). Descriptive statistics were calculated for all variables. Associations between antimicrobial resistance outcomes and animal species were assessed using chi-square (χ^2^) tests in R. For 2 × 2 comparisons, Yates continuity correction was applied consistent with the default behavior of the chisq.test() function in R, while larger contingency tables were analyzed without continuity correction. Statistical significance was set at *p* ≤ 0.05.

Principal Component Analysis (PCA) was conducted using the prcomp() function in R on centered and scaled antimicrobial resistance variables. Antimicrobial susceptibility outcomes were coded numerically as resistant = 1, intermediate = 0.5, and susceptible = 0. Principal components were evaluated using eigenvalues, scree plot inspection, and interpretability of component loadings. The first four principal components were retained for descriptive interpretation, although only PC1 and PC2 met the Kaiser criterion of eigenvalue >1.

Binary logistic regression analysis was performed using generalized linear models (glm function; family = binomial) to evaluate the association between animal species and multidrug resistance (MDR) status. MDR was defined as resistance to three or more antimicrobial classes. Predictor variables included animal species together with *blaCTX-M* and *blaSHV* gene carriage status. Cattle isolates were used as the reference category for species comparisons. Odds ratios (ORs) and corresponding 95% confidence intervals (CIs) were derived from model coefficients. Exploratory multivariable models incorporating individual resistance phenotypes and ESBL gene carriage were evaluated but were not retained because of collinearity and sparse-data instability.

## 3 Results

### 3.1 Isolation rate

Of 100 rectal swabs collected, 94 (94.0%) yielded confirmed *E. coli* isolates by MALDI-TOF MS: 48 from pigs and 46 from cattle. Six samples (two pigs, four cattle) yielded no bacterial growth and were excluded from all further analyses.

### 3.2 Antimicrobial resistance profiles

**Figure 1:**
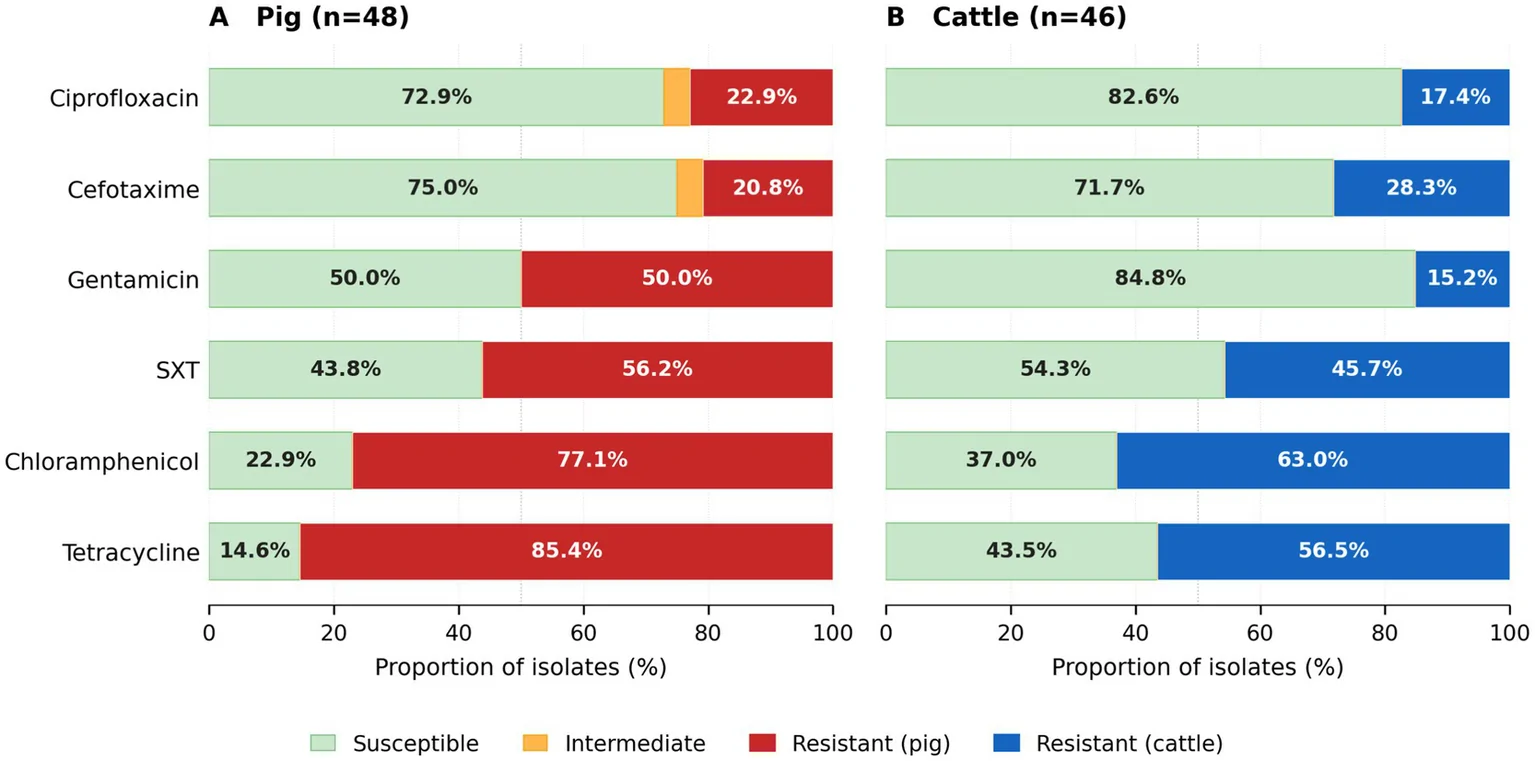
Antimicrobial susceptibility profiles of *Escherichia coli* isolates from pigs **(A)** (*n* = 48) and cattle **(B)** (*n* = 46) across six antibiotic classes. Bars show the proportion of isolates classified as susceptible, intermediate, or resistant. Percentage labels indicate the resistant fraction where ≥8%. SXT, sulfamethoxazole-trimethoprim.

Resistance rates differed across antibiotic classes and between species (Table 2; Figure 1). Pig isolates showed significantly higher resistance than cattle isolates to gentamicin (50.0% vs. 15.2%; χ^2^ = 11.332, *p* = 0.0008) and tetracycline (85.4% vs. 56.5%; χ^2^ = 8.220, *p* = 0.004). Chloramphenicol resistance was high in both species (77.1% vs. 63.0%; χ^2^ = 1.593, *p* = 0.207). Most isolates from both species remained susceptible to ciprofloxacin and cefotaxime. Resistance to sulfamethoxazole-trimethoprim did not differ significantly by species (56.2% pigs vs. 45.7% cattle; *p* = 0.412).

**Table 2:** Antimicrobial resistance profiles of *E. coli* isolates from pigs and cattle.

Parameter	Gentamicin	Chloramphenicol	Ciprofloxacin	SXT	Cefotaxime	Tetracycline
Pig resistant, n (%)	24 (50.0)	37 (77.1)	11 (22.9)	27 (56.2)	10 (20.8)	41 (85.4)
Pig susceptible, n (%)	24 (50.0)	11 (22.9)	35 (72.9)	21 (43.8)	36 (75.0)	7 (14.6)
Pig intermediate, n (%)	0 (0)	0 (0)	2 (4.0)	0 (0)	2 (4.0)	0 (0)
Cattle resistant, n (%)	7 (15.2)	29 (63.0)	8 (17.4)	21 (45.7)	13 (28.3)	26 (56.5)
Cattle susceptible, n (%)	39 (84.8)	17 (37.0)	38 (82.6)	25 (54.3)	33 (71.7)	20 (43.5)
Cattle intermediate, n (%)	0 (0)	0 (0)	0 (0)	0 (0)	0 (0)	0 (0)
χ^2^	11.332	1.593	2.556	0.674	2.480	8.220
*p*-value	0.0008**	0.207 (NS)	0.279 (NS)	0.412 (NS)	0.289 (NS)	0.004**

SXT, sulfamethoxazole-trimethoprim; NS, not significant; **p < 0.01.

### 3.3 Multidrug resistance

**Figure 2:**
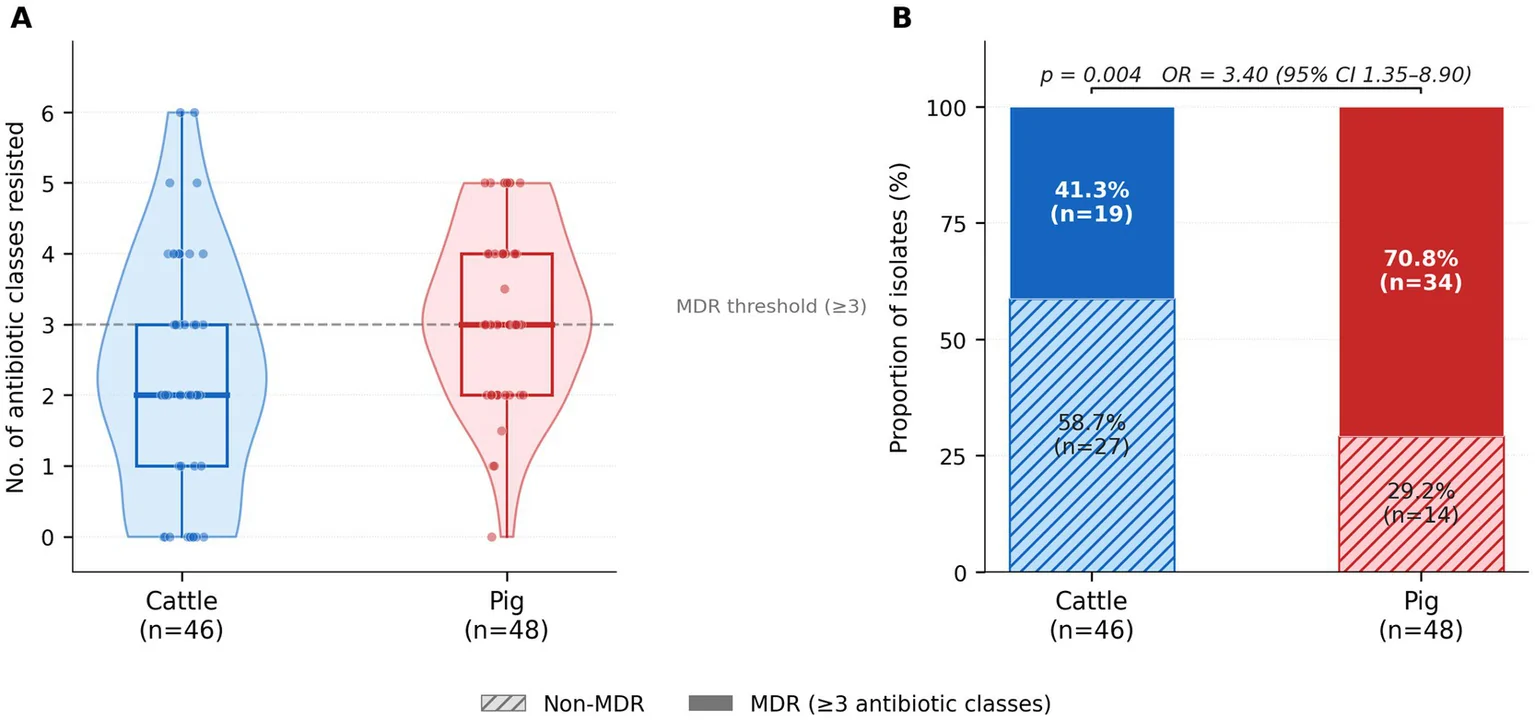
Multidrug resistance (MDR) distribution by species (pig *n* = 48; cattle *n* = 46). **(A)** Violin plot with overlaid box-and-whisker and individual data points showing the number of antibiotic classes resisted per isolate; The dashed line marks the MDR threshold (≥3 classes). **(B)** Proportional stacked bar chart of MDR vs. non-MDR isolates by species, with statistical annotation.

MDR was significantly more prevalent in pig isolates (70.8%, 34/48) than in cattle isolates (41.3%, 19/46; crude OR = 3.40, 95% CI 1.35–8.90; p = 0.004). Pigs exhibited a mean of 3.17 antibiotic classes resisted per isolate, compared with 2.26 in cattle (Table 3; Figure 2). This crude, species-only odds ratio differs from the adjusted odds ratio (3.97) derived from the multivariable logistic regression model reported in Section 3.6, which additionally accounts for *blaCTX-M* and *blaSHV* carriage; both estimates indicate a consistent, significantly higher MDR burden in pig isolates.

**Table 3:** Multidrug resistance summary by species.

Species	MDR (≥3 drugs), n (%)	Mean drugs resisted	Odds ratio (95% CI)	*p*-value
Pig (*n* = 48)	34 (70.8)	3.17	3.40 (1.35–8.90)	0.004**
Cattle (*n* = 46)	19 (41.3)	2.26	Reference	–

MDR, multidrug resistance (resistance to ≥3 of 6 antibiotic classes). **p < 0.01.

### 3.4 ESBL gene prevalence

**Figure 3:**
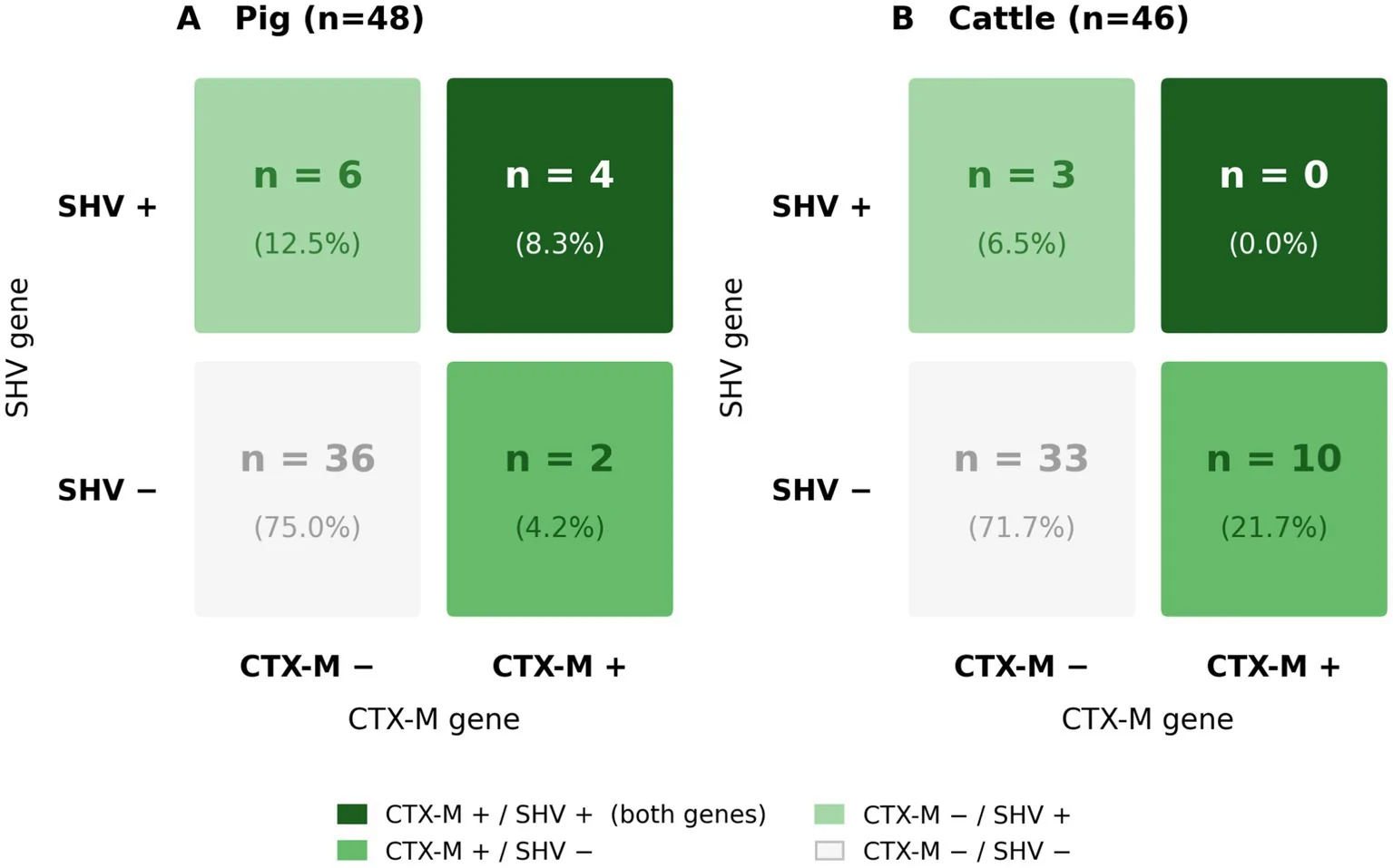
Descriptive ESBL gene co-carriage pattern (*blaCTX-M* x *blaSHV*) in pig **(A)** (*n* = 48) and cattle **(B)** (*n* = 46) isolates. Each cell shows the number and percentage of isolates carrying each gene combination. Color intensity indicates higher cell counts.

*blaCTX-M* was detected in 6 pig isolates (12.5%) and 10 cattle isolates (21.7%); *blaSHV* was detected in 10 pig isolates (20.8%) and 3 cattle isolates (6.5%). Four pig isolates carried both genes, compared with none in cattle; given the small numbers involved, this co-carriage finding should be interpreted with caution. Overall, 25 of 94 isolates (26.6%) harbored at least one ESBL gene (12 pig, 13 cattle). Differences were not statistically significant for either gene (*blaCTX-M*: χ^2^ = 1.420, *p* = 0.234; *blaSHV*: χ^2^ = 2.926, *p* = 0.087) (Table 4; Figure 3). The isolates which were negative for *blaCTX-M* and *blaSHV* were classified as negative for these two specific genes; however, they may still harbor other untested ESBL, AmpC, or beta-lactam resistance determinants not targeted in this study. The detection of *blaCTX-M* genes strongly indicates ESBL production, however, it should be noted that not all *blaSHV* variants confer extended-spectrum activity. PCR detection of *blaSHV* needs further sequencing to differentiate between narrow-spectrum and ESBL variants. Therefore, the findings of *blaSHV* carriage should be interpreted with caution, acknowledging that limitation.

**Table 4:** Prevalence of ESBL resistance genes in *E. coli* isolates from pigs and cattle.

Species	*blaCTX-M* positive, n (%)	*blaSHV* positive, n (%)	Any targeted ESBL gene, n (%)
Pig (*n* = 48)	6 (12.5)	10 (20.8)	12 (25.0)
Cattle (*n* = 46)	10 (21.7)	3 (6.5)	13 (28.3)
Total (*n* = 94)	16 (17.0)	13 (13.8)	25 (26.6)
χ^2^	1.420	2.926	0.128
*p*-value	0.234 (NS)	0.087 (NS)	0.721 (NS)

NS, not significant (*p* > 0.05). Any targeted ESBL gene, isolate positive for blaCTX-M and/or blaSHV. Chi-square and *p*-values reflect between-species comparisons for each gene category.

### 3.5 Principal component analysis

**Figure 4:**
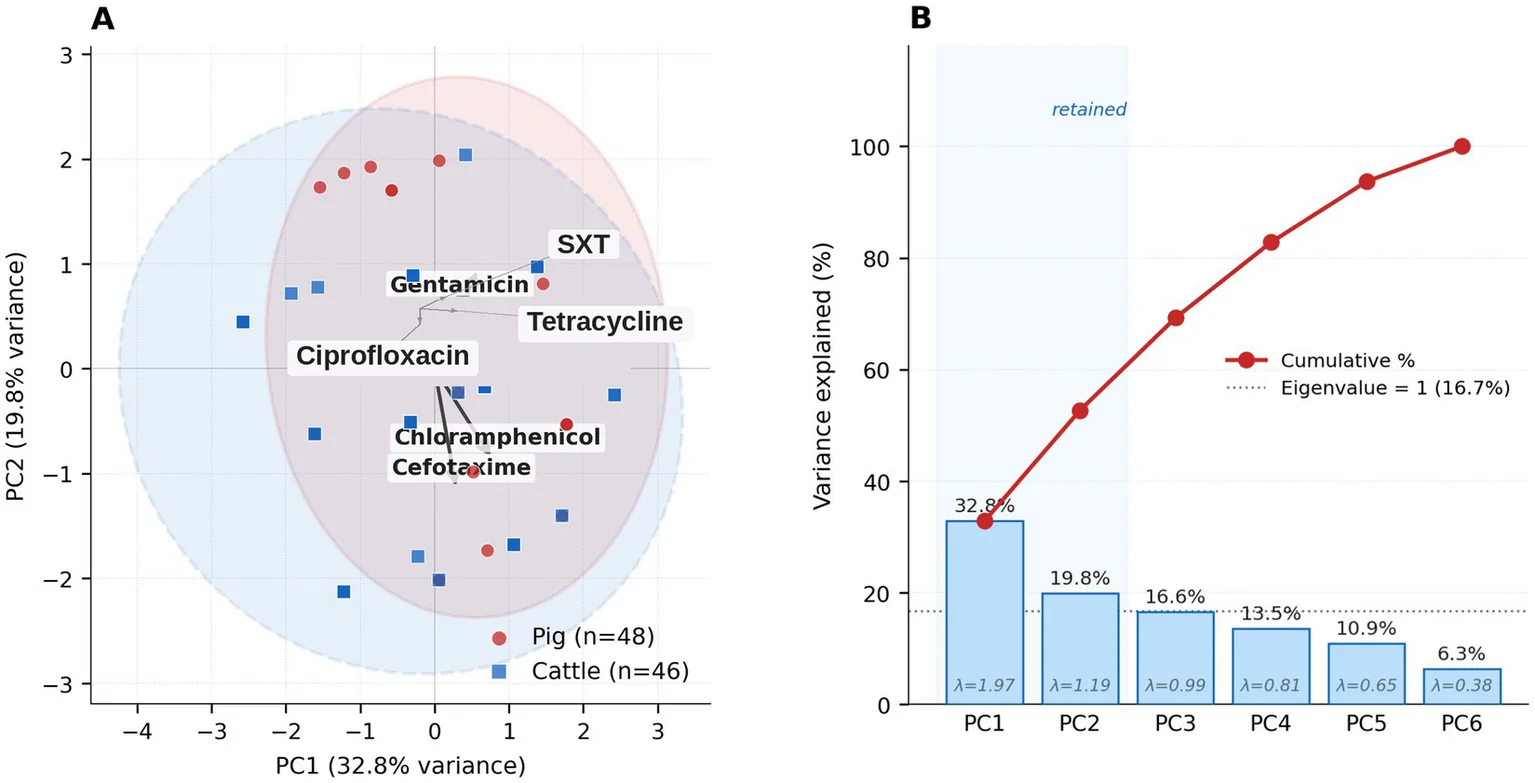
Principal Component Analysis (PCA) of antimicrobial resistance profiles. **(A)** Biplot of PC1 versus PC2 scores by species with 90% confidence ellipses, showing partial overlap between pig and cattle isolate distributions. Arrows indicate the direction and relative contribution of antimicrobial resistance variables. **(B)** Scree plot showing variance explained by each principal component and cumulative variance across components. Results are presented for exploratory visualization of resistance co-structure only and do not establish co-selection or underlying resistance mechanisms. GM, gentamicin; CHL, chloramphenicol; CIP, ciprofloxacin; SXT, sulfamethoxazole-trimethoprim; CTX, cefotaxime; TET, tetracycline.

Principal Component Analysis (PCA) of antimicrobial resistance profiles was performed to explore patterns of co-occurring resistance among isolates and to reduce dimensionality within the resistance dataset (Figure 4). The first two principal components explained 52.7% of total variance across the six antimicrobial resistance variables. PC1 accounted for 32.8% of total variance and was primarily associated with tetracycline, sulfamethoxazole-trimethoprim, and chloramphenicol resistance, indicating a broad multidrug resistance pattern involving commonly used antimicrobials. PC2 explained 19.8% of variance and reflected variation involving gentamicin and cefotaxime resistance, suggesting differences between aminoglycoside-associated and cephalosporin-associated resistance profiles.

The PCA biplot demonstrated partial separation between pig and cattle isolates, with pig isolates tending toward higher PC1 scores, consistent with the greater multidrug resistance burden observed among pig isolates in univariate analyses. However, substantial overlap between pig and cattle isolate distributions was observed, indicating that resistance profiles were only partially differentiated by species. The direction and magnitude of the loading vectors further illustrated relationships among antimicrobial resistance variables, with tetracycline, sulfamethoxazole-trimethoprim, and chloramphenicol resistance showing similar directional contributions to PC1, suggesting co-occurrence of these resistance traits within isolates. Overall, the PCA findings should be interpreted as exploratory descriptions of resistance co-structure rather than evidence of distinct species-specific clustering.

### 3.6 Logistic regression

Multivariate logistic regression identified animal species as a significant predictor of MDR. Pig isolates had significantly higher odds of MDR compared with cattle isolates (OR = 3.97, 95% CI 1.66–9.51; *p* = 0.002) (Table 5). This indicates that isolates recovered from pigs were nearly four times more likely to exhibit multidrug resistance than those recovered from cattle. In contrast, neither *blaCTX-M* nor *blaSHV* gene carriage showed a statistically significant association with MDR status (*p* > 0.05). The absence of a significant association between ESBL gene carriage and MDR suggests that phenotypic multidrug resistance within the study population may involve additional resistance mechanisms beyond the ESBL-associated genes investigated. Overall, the regression model supports the observation that livestock production system differences, particularly those associated with pig production, may play an important role in shaping antimicrobial resistance burden within slaughter animal populations.

**Table 5:** Logistic regression model for predictors of multidrug resistance (MDR).

Predictor	β	Odds ratio (OR)	95% CI lower	95% CI upper	*p*-value
Pig isolates	1.378	3.97	1.66	9.51	0.002**
CTX-M positive	−0.111	0.90	0.29	2.81	0.849
SHV positive	−0.958	0.38	0.10	1.48	0.164

Outcome variable: MDR status (1 = MDR; 0 = non-MDR) reference category: cattle isolates MDR definition: resistance to ≥3 antimicrobial classes tested. **p < 0.01.

## 4 Discussion

This study provides the first comparative characterization of *E. coli* harboring targeted ESBL-associated genes from pigs and cattle slaughtered at urban abattoirs in Lilongwe District, Malawi. By integrating phenotypic antimicrobial susceptibility testing, genotypic ESBL detection, and multivariate statistical analyses, the study identified important interspecies differences in antimicrobial resistance patterns and MDR burden. The findings demonstrate that pig isolates carried a substantially higher MDR burden than cattle isolates and exhibited significantly greater resistance to gentamicin and tetracycline.

Gentamicin resistance was significantly higher among pig isolates (50.0%) compared with cattle isolates (15.2%). Similar findings have been reported in Tanzania (24) and Estonia (17), where aminoglycoside resistance was more prevalent in swine-associated isolates than in bovine isolates. These differences likely reflect variations in production systems and antimicrobial usage practices between livestock species. Pig production systems are frequently characterized by more intensive management and greater antimicrobial exposure, which may generate stronger selective pressure for resistant bacteria. Tetracycline resistance was also markedly elevated in pigs (85.4%) relative to cattle (56.5%), consistent with previous reports from sub-Saharan Africa and Europe (24, 25). Tetracyclines are among the most widely used antimicrobials in livestock production because of their affordability and broad availability, and their prolonged use may contribute to persistence of resistance genes within animal production systems.

Chloramphenicol resistance was high in both species without significant interspecies differences. Although chloramphenicol use has declined in many countries, resistance determinants may persist due to historical antimicrobial exposure and co-selection with other resistance genes located on shared mobile genetic elements. The PCA suggested co-occurring resistance patterns involving tetracycline, sulfamethoxazole-trimethoprim, and chloramphenicol within the major resistance component, suggesting co-occurrence of resistance traits. However, the overlap observed between pig and cattle isolates in the PCA biplot indicates that resistance patterns were only partially separated between species. Therefore, the PCA findings should be interpreted as exploratory descriptions of resistance structure rather than evidence of distinct species-specific clustering.

Ciprofloxacin and cefotaxime remained effective against most isolates from both species, although emerging resistance was observed, particularly among pig isolates. The relatively lower prevalence of cefotaxime resistance compared with tetracycline and chloramphenicol resistance may reflect lower exposure to third-generation cephalosporins in local livestock production systems. Nevertheless, the detection of cefotaxime-resistant isolates and ESBL-associated genes remains a significant public health concern because these antimicrobials are critically important in human medicine. One of the most important findings of this study was the significantly higher prevalence of MDR among pig isolates compared with cattle isolates (70.8% vs. 41.3%). Logistic regression analysis further demonstrated that pig isolates had approximately fourfold higher odds of MDR than cattle isolates. This finding supports the hypothesis that species-specific production systems and antimicrobial usage practices contribute substantially to differences in resistance burden. Intensive pig farming systems may facilitate persistence and dissemination of multidrug-resistant bacteria through repeated antimicrobial exposure, close animal confinement, and environmental contamination.

Although targeted ESBL-associated genes were detected in isolates from both species, neither *blaCTX-M* nor *blaSHV* carriage was significantly associated with MDR status in the logistic regression model. This suggests that phenotypic multidrug resistance within the study population may be influenced by additional resistance mechanisms beyond the two targeted ESBL-associated genes investigated. Resistance determinants such as efflux pumps, integrons, plasmid-mediated resistance genes, and chromosomal mutations may contribute to the MDR profiles observed. These results raise important implications for surveillance, highlighting the value of combining phenotypic antimicrobial susceptibility testing with molecular detection methods in antimicrobial resistance surveillance programs. It is important to note that direct comparisons of MDR prevalence across countries are limited by differences in sampling frames, production systems, antimicrobial usage practices, and laboratory methodologies.

The prevalence of *blaCTX-M* detected in this study was lower than levels reported in some Asian countries, including China (26), where intensive livestock production systems and higher antimicrobial usage have been associated with widespread dissemination of ESBL-producing bacteria. In contrast, the *blaSHV* prevalence observed among pig isolates was comparable to reports from Brazil and parts of Europe (27, 31). Differences between studies may reflect variation in antimicrobial usage practices, livestock management systems, surveillance intensity, and circulating plasmid populations across geographical regions.

The findings of this study have important implications within the One Health framework. Urban abattoirs represent critical interfaces linking animal reservoirs, food products, workers, and the wider human population. Resistant *E. coli* originating from food animals may contaminate carcasses and meat products during slaughter and processing, thereby facilitating dissemination through the food chain. In addition, disposal of animal waste and abattoir effluent may contribute to environmental dissemination of resistant bacteria. The overlap between resistance profiles reported in livestock and those increasingly described in human clinical isolates across sub-Saharan Africa suggests potential transmission pathways between animal and human populations. Strengthening antimicrobial stewardship in livestock production, improving hygiene practices within slaughter systems, and implementing systematic AMR surveillance at abattoirs are therefore essential components of national One Health AMR control strategies.

This study has several limitations. The sample size limited the statistical power for subgroup analyses and reduced precision for some regression estimates. Only two ESBL-associated gene families (*blaCTX-M* and *blaSHV*) were investigated, excluding other clinically important resistance genes such as *blaTEM*, *blaOXA*, and carbapenemase-associated genes. The cross-sectional design also limits inference regarding temporal dynamics and sources of resistance acquisition. Furthermore, whole-genome sequencing and plasmid characterization were not performed, restricting detailed analysis of resistance mechanisms and transmission pathways. Future studies incorporating larger sample sizes, genomic epidemiology, and longitudinal surveillance approaches would provide a more comprehensive understanding of antimicrobial resistance dissemination within livestock production systems in Malawi.

## 5 Conclusion

*Escherichia coli* harboring targeted ESBL-associated genes are present in both pigs and cattle at urban abattoirs in Lilongwe District, Malawi. Pig isolates carry a significantly higher MDR burden (70.8% vs. 41.3%; adjusted OR = 3.97, 95% CI 1.66–9.51, *p* = 0.002), with significantly higher gentamicin and tetracycline resistance observed among pig isolates. Tetracycline and chloramphenicol are largely ineffective against isolates from both species. Ciprofloxacin and cefotaxime remain effective but show emerging resistance trends requiring continued monitoring. PCA revealed co-selected broad-spectrum resistance (PC1: 32.8%) and a distinct aminoglycoside-cephalosporin axis (PC2: 19.8%). Neither *blaCTX-M* nor *blaSHV* carriage significantly predicted MDR status, a finding consistent with the fact that both genes confer resistance within a single antibiotic class (*β*-lactams), whereas MDR was defined as resistance to three or more of the six antibiotic classes tested; screening for *blaCTX-M* and *blaSHV* alone cannot, therefore, be expected to predict MDR status; broader genotypic screening (e.g., additional resistance-gene targets or whole-genome sequencing) is needed to capture the mechanisms underlying multidrug resistance. This underscores the need for combined phenotypic and genotypic surveillance to provide full information of resistance status. These findings underscore the urgent need for evidence-based antibiotic stewardship in Malawian livestock production and systematic AMR monitoring at urban abattoirs within a One Health framework.

## Data Availability

The original contributions presented in the study are included in the article/Supplementary material, further inquiries can be directed to the corresponding author.
